# Relationship Between Parental Marital Conflict and Social Anxiety Symptoms of Chinese College Students: Mediation Effect of Attachment

**DOI:** 10.3389/fpsyg.2021.640770

**Published:** 2021-09-06

**Authors:** Aklilu A. Adare, Yuewen Zhang, Yaqi Hu, Zhenhong Wang

**Affiliations:** Shaanxi Provincial Key Research Center of Child Mental and Behavioral Health, School of Psychology, Shaanxi Normal University, Xi' an, China

**Keywords:** social anxiety, parental attachment, peer attachment, perceived parental marital conflict, Chinese college students

## Abstract

Social anxiety has been a common problem among college students and has an adverse impact on their adaptation outcomes. Among influential factors, parental marital conflict and attachment (parental and peer attachment) have been found to be related to social anxiety symptoms of college students; however, little is known how parental marital conflict and attachment jointly contribute to social anxiety symptoms of college students. The current study explored this issue. Self-reported questionnaires of perception of children of interparental conflict scale, inventory of parent and peer attachment, and the social interaction anxiety scale were administered to 707 undergraduate students (Mean age = 19.27, *SD* = 0.97). Results indicated that perceived parental marital conflict was positively correlated with social anxiety symptoms and was negatively associated with parental and peer attachment. Parental and peer attachments were negatively correlated with social anxiety symptoms. Mediation analyses indicated that perceived parental marital conflict exerted its indirect effect on social anxiety symptoms through a serial multiple mediation role of parental and peer attachment. The present findings highlight the serial multiple mediation role of parental and peer attachment in the relationship between perceived parental marital conflict and social anxiety symptoms of college students.

## Introduction

Recently, the psychological well-being of college students has been a wide concern for society. Among the multiple physical and mental problems, social anxiety has become one of the major psychological problems of college students (Zhao and Dai, 2016; Shi et al., 2019). Social anxiety refers to the negative emotional experience like tension, uneasiness, and fear of social situations caused by the excessive worry of being evaluated or scrutinized by others in public (Morrison and Heimberg, 2013; Boehme et al., 2014), which has an adverse impact on academic, social, and emotional functioning of college students (Book and Randall, 2002; Auerbach et al., 2018; Jia et al., 2019; Zhang et al., 2019). Previous studies showed that the level of social anxiety increased significantly from adolescence to early adulthood, especially in college years, during which various challenges (i.e., interpersonal communication problems) need to be faced which might more likely lead to higher levels of social anxiety symptoms than other age groups (Herman, 1998). Multiple factors contribute to the development of social anxiety symptoms of college students (El-Sheikh et al., 2013; Jia et al., 2019; Zhang et al., 2019), among which family risk environmental factors such as parental marital conflict (Riggio, 2004; Fosco and Feinberg, 2015) has been regarded as an important contributor, and perceptions about such conflicts may be a key factor linking marital dissatisfaction with deleterious adaptations like social anxiety symptoms (Cummings and Schatz, 2012). In addition, attachment (parental and peer attachment) has been found to be associated with social anxiety symptoms of college students (Lu et al., 2015; Gorrese, 2016; Manes et al., 2016). However, previous studies focused on the relation between perceived parental marital conflict, parental and peer attachment, and social anxiety symptoms mostly on Western children and adolescents, while research investigating Chinese college students is still lacking. Therefore, the current study aimed to explore whether parental and peer attachment played a serial multiple mediation role in the relationship between perceived parental marital conflict and social anxiety symptoms in Chinese college students.

### Parental Marital Conflict and Social Anxiety

Parental marital conflict refers to any disagreement, difference, or argument regarding an issue of family life, which includes all kinds of physical and psychological conflicts, and has been commonly considered as a core predictor of family solidarity and the key factor in determining family life quality (Erel and Burman, 1995; Cummings and Davies, 2002). Emotional security theory (EST, Davies and Cummings, 1994), as an important theoretical model to investigate the impact of parental marital conflict on the adaptation of children, has been supported by numerous empirical studies (e.g., Kouros et al., 2010; Cummings et al., 2013). A core perspective of EST is that the internalized representations of family relations and response processes of children that develop with time have profound effects on their long-term adaptation (Davies and Cummings, 1994), and perceptions that children have about parental marital conflict may be a key factor linking marital dissatisfaction with maladaptations of the children. According to the EST, perceptions about parental marital conflict destroy the emotional security of children about family relations, increases their negative behavioral and emotional responses, thus increasing their psychological maladaptation (Davies and Cummings, 1994). Based on EST, a large number of studies have shown that perceived parental marital conflict is associated with adjustment problems of children and adolescents like externalizing problems, internalizing problems, and academic difficulties (Davies and Lindsay, 2004; Cummings and Davies, 2010; Cummings et al., 2012; for reviews, see Grych and Fincham, 2001; Li Y. et al., 2016; Khurshid et al., 2019; Li D. et al., 2020). Specifically, empirical studies provided evidence that perceived parental marital conflict could impact social anxiety symptoms (Riggio, 2004; Gao et al., 2018) of individuals. For example, Riggio (2004) found that perceived parental marital conflict had a significant effect on the anxiety of young adults in personal relationships.

### Attachment and Social Anxiety

Attachment refers to a close, long-lasting emotional bond between an infant and a caregiver (Bowlby, 1969/1982), or a long-lasting emotional bond of substantial intensity (Armsden and Greenberg, 1987). Research of attachment is based on two theoretical viewpoints, that is, internal working models (IWMs) and attachment relationship approach (Buist et al., 2002, 2004; Li J. B. et al., 2020). The two viewpoints are different but not contradicting in understanding attachment to adolescents and their developmental adaptations. The most significant difference between these two perspectives is how attachment is conceptualized. The IWM is noted as the social cognitive perspective and is largely focused on the assessment of mental representations of people of both the self and others in close relationships, which reflects the relative stability and continuity of attachment (Bartholomew, 1990). According to the attachment relationship approach, attachment is supposed to emphasize specific relationships and is seen as changeable throughout time. As adolescents grow up, the security fostered by their parents becomes more dependent on the abilities of their parents to function as competent allies and less on their actual presence (i.e., availability; Weiss, 1982; Armsden and Greenberg, 1987), which means that the maturity of adolescents and its related adaptations might be associated with greater attachment represented as a parent-child relationship instead of the one represented as the actual availability of parents. Therefore, researchers suggested that investigating specific attachment relationships could help better explain the function and development of attachment in early and later adolescence than IWMs (Buist et al., 2002; Li D. et al., 2020). Furthermore, a self-report measure, inventory of parent and peer attachment (IPPA) (Armsden and Greenberg, 1987; Li D. et al., 2020), is commonly used to assess specific attachment relationships including parental and peer attachment in youth (Armsden and Greenberg, 1987). Accordingly, the measure of IPPA was used in the current study focusing on the specific attachment of later adolescent (i.e., college students) relationships with their parents and peers.

Parental attachment is a tight emotional bond established between the infant and parents (Bowlby, 1969/1982). The quality of parental attachment relationships is significantly related to the social adaptation and development of individuals (Li C. et al., 2016). Regarding social anxiety symptoms, previous research suggested that parental attachment and social anxiety symptoms are intensely intertwined and that insecure and dysfunctional parental attachment relationships may predispose adolescents to social anxiety symptoms (Leung et al., 1994; Eng et al., 2001; Harvey et al., 2005). Therefore, a low-quality parental attachment relationship is another important factor that influences the development and maintenance of social anxiety symptoms (Mothander and Wang, 2014; Zhao et al., 2014; Liu et al., 2017).

In addition, recent studies have emphasized the impact of peer attachment relationships on social anxiety symptoms. Peer attachment is defined as a significant and enduring emotional and social bond with close friends, which is characterized by good communication, mutual understanding, trust, and emotional closeness (Laible et al., 2000; Theisen et al., 2018). Adolescents who are insecurely attached to their peers perceive their close friends as neither supportive nor trustworthy and often feel rejected by their close friends, and thus tend to develop social anxiety symptoms (Gorrese, 2016). A low-quality peer attachment relationship is also one of the robust predictors of social anxiety symptoms among college students (Burge et al., 1997). During college years, low-quality peer attachment relationships such as lack of intimacy, companionship, emotional support, and closeness are associated with increased social anxiety symptoms (Greca and Lopez, 1998; Hawker and Boulton, 2000).

### Parental Marital Conflict, Attachment, and Social Anxiety Symptoms

Attachment theory highlights the significance of security in the attachment relationship as an internal goal for human beings and lays the groundwork for the future interpersonal relationship quality, whereas EST postulates that maintaining safety and security in the interpersonal relationships are among the most salient in the hierarchy of human goals (Bowlby, 1973; Davies and Woitach, 2008). The EST literature showed that parental marital conflict may spill over to parenting behaviors and attachment relationships to influence the emotional insecurity and adjustment of the individual (e.g., Schermerhorn et al., 2008). Based on EST (Davies and Cummings, 1994; Davies et al., 2002) and the attachment relationship approach (Buist et al., 2002, 2004; Li J. B. et al., 2020), individuals might feel insecure and seem to fear parents and/or be away from them when they perceive parental marital conflict, which in turn can influence their attachment quality. Previous evidences have verified the link between perceived parental marital conflict and attachment (Stocker and Youngblade, 1999; El-Sheikh and Elmore-Staton, 2004; Yang et al., 2016). For example, Yang et al. (2016) found that a higher level of perceived parental marital conflict was negatively linked to the attachment of college students including parental attachment and peer attachment. Thus, it can be assumed that perceived parental marital conflict was negatively associated with the attachment quality of college students toward parents and peers.

In addition, parental and peer attachment are closely related. Attachment theory proposes that attachment relationships initially begin with parents and their infants and serve as affective support and a safe base. As individuals grow up, attachment relationships expand toward peers, and such relationships become the central arena in which attachment processes are possible to play out during adolescence and beyond and contribute to multiple aspects of psychosocial adaptations (Ainsworth, 1989). The positive relationship of parental and peer attachment has been verified, and the mediation role of peer attachment linking parental attachment to adaptation performance of individuals (i.e., addictive behaviors; positive social adjustment, and anxiety) has also been confirmed previously (Gorrese, 2016; Yang et al., 2016; Davies et al., 2018). Furthermore, as noted before, poor attachment relationship quality has been regarded as a risk factor contributing to maladjustments of individuals, such as internet addiction (Deng et al., 2013a,b; Yang et al., 2016) and social anxiety symptoms (Wu and Wang, 2014; Lu et al., 2015; Gorrese, 2016; Manes et al., 2016; Pan et al., 2016; Manning et al., 2017; Yu et al., 2019). Based on the theories and previous findings, it could be assumed that parental and peer attachment relationships were significantly related to social anxiety symptoms of college students.

As outlined above, both parental and peer attachment have been regarded as important mediators linking parental marital conflict to adaptations of college students. However, the previous researches were mainly conducted among Western children and adolescents, with only a few studies examining the effects of parental and peer attachment between perceived parental marital conflict and social anxiety symptoms based on Chinese college students.

### The Present Study

**Figure 1:**
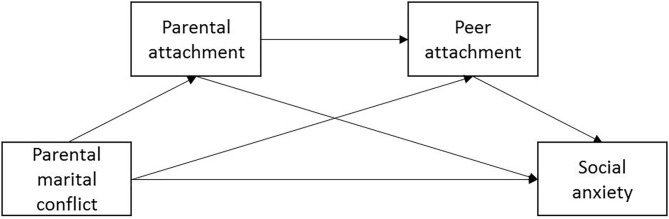
The conceptual framework of the serial multiple mediation model.

In summary, guided by EST (Davies and Cummings, 1994) and attachment theory (Buist et al., 2002, 2004; Li D. et al., 2020), the current study aimed to examine the serial multiple mediation role of parental and peer attachment in the relationship between perceived parental marital conflict and the social anxiety symptoms of Chinese college students. Based on the literature reviewed above, we hypothesized that (1) perceived parental marital conflict would be positively associated with social anxiety symptoms and negatively associated with attachment quality (including parental and peer attachment). Both parental and peer attachment would be negatively associated with social anxiety symptoms, and (2) the association between perceived parental marital conflict and social anxiety symptoms would be mediated by parental and peer attachment (see the hypothesized mediation model, Figure 1).

## Methods

### Participants

Only Chinese college students were eligible to participate in the present study. We recruited participants from Xi'an, China, using convenience sampling techniques, and a total of 707 college students (133 males, mean age = 19.27 years, SD = 0.97, age range: 16–25) participated in the present study. Among them, 50.4% of the students were from urban areas, 28% from the town, and 21.6% from rural areas. The sample consisted of four grades: 18.8% were freshmen, 44.6% were sophomores, 28.7% were juniors, and 7.9% were seniors. In addition, 33.9% of the students came from the fields of liberal arts, and 66.1% came from the fields of science.

About 83.7% of the participants reported that their average monthly household income was <10,000 RMB. For parental education levels, 64.1% of the participants reported that their father had a high school degree or less and 34.4% of the fathers had a bachelor's degree or above. Almost 71.1% of the participants reported that their mother had a high school degree or less and 28.1% of the mothers had a bachelor's degree or above.

### Procedure

The present study was approved by the Academic Ethics Committee of the School of Psychology, Shaanxi Normal University, China. Informed consent was obtained prior to data collection. The study consisted of two parts. First, a total of 230 questionnaires were distributed by two researchers in classrooms, and 225 valid questionnaires were returned. To minimize the impact of social desirability, each individual was informed by the researcher to answer every question on the questionnaires as honestly as possible. Second, to ensure an optimal sample size, researchers collected another 482 questionnaires online. Based on the requirement that no item on the online survey should have missing data, all responses of the 482 college students were included in the present study. Thus, there were a total of 707 valid questionnaires in this study.

### Measures

#### Parental Marital Conflict

Perceived parental marital conflict was measured using the Chinese version of children's perception of interparental conflict scale (CPIC; Grych et al., 1992) revised by Chi and Xin (2003). This scale has shown good reliability and validity among Chinese college students (Qing et al., 2017). This study only adopted the subscale of conflict property which had 19 items, including conflict frequency (six items), conflict intensity (seven items), and conflict resolution (six items) [χ^2^/df = 5.13, tucker-lewis index (TLI) = 0.91, comparative fit index (CFI) = 0.92, root-mean-square error of approximation (RMSEA) = 0.074]. All items were rated on a 4-point Likert scale (from 1 = not at all compliant to 4 = completely compliant). The higher scores indicated higher levels of perceived parental marital conflict. For the current study, Cronbach's alpha for the scale was 0.94.

#### Attachment

An attachment was assessed using the inventory of parent and peer attachment (IPPA; Armsden and Greenberg, 1989), which consisted of three forms for the mother, father, and peer-yielding three attachment scores, each with 25 items. The scale had three subscales: trust, communication, and alienation. Participants were required to rate the items from 1 (almost never or never true) to 5 (almost always or always true). After the alienation items and the negatively worded items were reversely scored, the parental attachment (χ^2^/df = 5.13, TLI = 0.98, CFI = 0.98, RMSEA = 0.077) score was the sum of the 50 items from the mother form and father form, and the peer attachment (χ^2^/df = 4.42, TLI = 0.91, CFI = 0.92, RMSEA = 0.069) score was the sum of the 25 items from the peer form. Previous studies have proved that IPPA had well-established content validity and was suitable for college students (Guarnieri et al., 2015; Lepp et al., 2016; Yang et al., 2016). In the current study, Cronbach's alpha coefficients were 0.86 and 0.85 for parent and peer attachment, respectively, and 0.89 for the total scale.

#### Social Anxiety Symptoms

The social interaction anxiety scale (SIAS; Mattick and Clarke, 1998) was administered to assess affective, behavioral, and cognitive reactions in 20 social interaction situations associated with interacting or engaging with others. It consisted of 20 items (χ^2^/df = 4.33, TLI = 0.91, CFI = 0.93, RMSEA = 0.069) such as “when mixing socially, I am uncomfortable” and “I have difficulty talking with other people,” each rated on a 5-point scale ranging from 0 (not at all characteristic of me) to 4 (extremely characteristic of me). This measure had shown good reliability and validity in samples of Chinese college students (Ye et al., 2007). Higher total scores indicated higher levels of social anxiety symptoms. Cronbach's α for the measure in this study was 0.90.

### Data Analysis

One researcher collated the data for analysis. The SPSS version 22.0 and AMOS 23.0 (IBM, Armonk, NY, USA) were used to conduct the following analyses. First, Pearson's correlation analysis was conducted to examine zero-order associations between all the study variables. Then, structural equation models (SEM) were developed based on hypothesized relationships between variables and tests of preliminary models. SEM is a very general, powerful multivariate technique that is used to explain the relationship between multiple variables and concepts, which can combine mediation analysis with latent variable analysis and provides model fit information about the consistency of the hypothesized mediation model to the data. Model fit was assessed by RMSEA, TLI, and CFI. The adequate fit was suggested for values less than or equal to 0.08 for the RMSEA and greater than or equal to 0.90 for the TLI and CFI (Hu and Bentler, 1999; Wen et al., 2004). Finally, the bootstrap method was used to test the indirect effects. We calculated bias-corrected and accelerated 95% bootstrap CIs based on 5,000 bootstrapped samples. If a 95% CI did not contain zero, then the indirect effect was significant. Specific indirect effects were estimated using an AMOS user-defined estimand.

## Results

### Common Method Bias

In the current study, all the data were collected using self-report measurements, which might lead to common method bias (Podsakoff et al., 2003). Thus, a single factor test of Harman was conducted to rule out the common method bias (Harman, 1976). According to Harman (1976), common method bias existed when only one factor emerged or when one factor explained for more than 40% of the variance associated with all items loaded simultaneously in factor analysis. The results of the factor analysis of the current study showed that a single factor explained 21.20% of the total variance, which indicated no significant common method bias.

### Descriptive Statistics and Correlation Analysis

Means, SDs, and bivariate correlations of all the study variables are displayed in Table 1. As hypothesized, perceived parental marital conflict was positively related to social anxiety symptoms, and negatively related to parental and peer attachment. Parental and peer attachments were negatively correlated with social anxiety symptoms. In addition, a positive relationship was found between parental attachment and peer attachment.

**Table 1:** Descriptive statistics and bivariate correlations for all study variables (*N* = 707).

	***M***	***SD***	**1**	**2**	**3**	**4**	**5**	**6**
1. Gender	–	–	–					
2. Age	19.27	0.97	−0.09^*^	–				
3. Perceived parental marital conflict	41.99	10.86	0.06	−0.00	–			
4. Parental attachment	55.12	14.79	0.03	−0.01	−0.53^***^	–		
5. Peer attachment	48.53	13.19	0.16^***^	−0.00	−0.21^***^	0.42^***^	–	
6. Social anxiety symptoms	45.54	12.42	−0.02	−0.01	0.26^***^	−0.38^***^	−0.34^***^	–

*N = 707, M, means; SD, standard deviation*;

* *p < 0.05*,

*** *p < 0.001, two-tailed*.

### Serial Multiple Mediation Analyses

**Figure 2:**
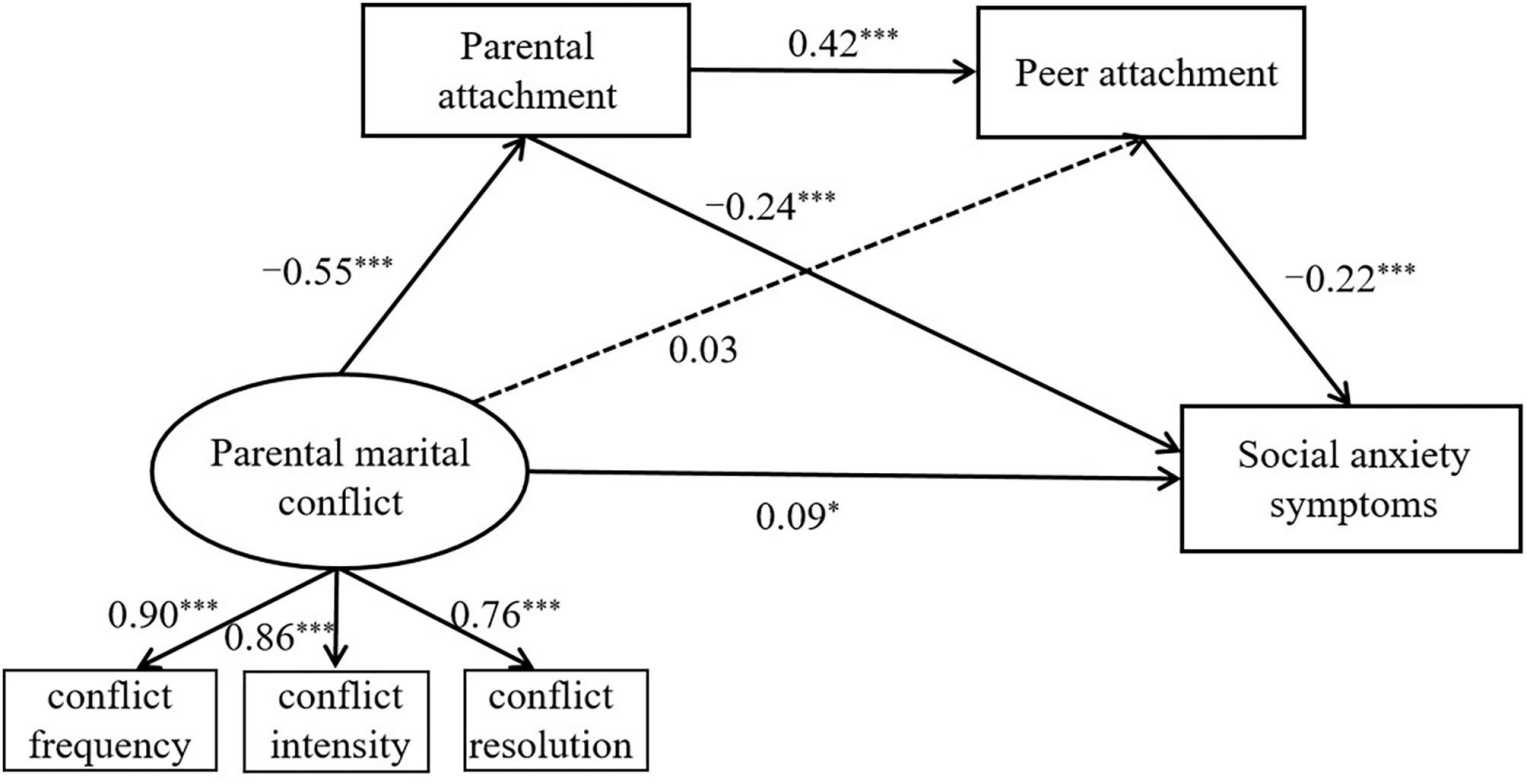
Standardized path coefficients for effects of parental marital conflict on social anxiety symptoms.

The direct and indirect effects of perceived parental marital conflict on social anxiety symptoms of college students were then examined using AMOS 23.0. We hypothesized that perceived parental marital conflict had a direct effect on social anxiety symptoms of college students, whereas perceived parental marital conflict influenced social anxiety symptoms of college students through serial multiple mediating effects of parental attachment and peer attachment. The fit indices indicated that the model (Model 1) was fitting well (χ^2^/df = 4.90, TLI = 0.97, CFI = 0.97, RMSEA = 0.074). However, in this model, the path from perceived parental marital conflict to peer attachment was not significant; we thus reexamined the model after the non-significant path had been deleted, and the goodness-of-fit for the final model (Model 2) showed that the model fitted the data well (χ^2^/df = 4.27, TLI = 0.97, CFI = 0.98, RMSEA = 0.068). Thus, Model 2 (path coefficients see Figure 2) demonstrated a better fit than Model 1 and could be used for further analysis. Finally, the bootstrap test showed that the serial multiple mediation effects of parental and peer attachment were significant in Model 2 (see Table 2). Thus, the perceived parental marital conflict of college students might exert an indirect effect on their social anxiety symptoms through parental attachment and peer attachment. Parental attachment and peer attachment could serially mediate the effect of perceived parental marital conflict on social anxiety symptoms of college students.

**Table 2:** Total and specific indirect effects for the final model.

**Model pathways**	**Estimate**	***SE***	**95% CI**	
			**Lower**	**Upper**
*Specific indirect effect*				
Perceived parental marital conflict → Parental attachment → Social anxiety symptoms	0.56	0.12	0.35	0.81
Perceived parental marital conflict → Peer attachment → Social anxiety symptoms	−0.03	0.05	−0.13	0.07
Perceived parental marital conflict → Parental attachment → Peer attachment → Social anxiety symptoms	0.21	0.05	0.13	0.30

*N = 707, SE, standard error; CI, confidence interval. All the values of point estimates were standardized*.

## Discussion

The current study aimed to investigate the mechanisms underlying the relation between perceived parental marital conflict and social anxiety symptoms of college students. Drawn from the perspectives of EST and attachment theory, we examined a serial multiple mediation model with parental and peer attachment in the association between perceived parental marital conflict and social anxiety symptoms among Chinese college students.

The present study found that perceived parent marital conflict was positively associated with social anxiety symptoms of college students. The family environment has been regarded as a particularly significant context for the social and psychological adjustment of individuals (Ko et al., 2015). According to EST (Davies and Cummings, 1994), a higher level of previous exposure to parental marital conflict left children primed for higher and even more negative, emotional responses in later conflict contexts. The present findings are in line with EST and empirical studies, thus suggesting that adolescents or college students who grow in families with chronic parental marital conflict are at a higher risk of acquiring and maintaining social anxiety symptoms (Riggio, 2004; Cusimano and Riggs, 2013; Gao et al., 2018). In addition, individuals who grow in families with frequent, hostile, and poorly resolved parental marital conflict have also been found to have internalizing and externalizing problems, such as substance use (Fosco and Feinberg, 2018), depression (Bernet et al., 2016; Tu et al., 2016), and decreased self-efficacy (Fosco and Feinberg, 2015). Altogether, it can be concluded that parental marital conflict, as an adverse family environmental factor, contributes to the maladjustment of individuals including social anxiety symptoms.

Consistent with the attachment relationship perspective (Buist et al., 2002, 2004; Li D. et al., 2020) and previous studies, the present study found that parental, peer attachment was negatively associated with social anxiety symptoms of college students. As mentioned previously, low-quality parental and peer attachment relationships could increase the risk of social anxiety problems (Mothander and Wang, 2014; Wu and Wang, 2014). Prior evidence has indicated that high-quality parental or peer attachment relationships could effectively cultivate and enhance interpersonal relationships of individuals and reduce the generation of social anxiety symptoms (Wang et al., 2017). On the contrary, individuals growing up in an environment of low-quality parental and peer attachment relationships might unconsciously learn from poor social models and are more likely to adopt maladaptive interpersonal strategies when interacting with others, resulting in interpersonal tensions and social anxiety symptoms (Wang et al., 2017). Accordingly, the present study finding suggests that college students with higher levels of parental or peer attachment relationships might have better interpersonal relationships and have lower levels of social anxiety symptoms. On the contrary, college students who have low-quality attachment relationships with their parents or peers might have maladaptive interpersonal skills in interacting with others and have higher levels of social anxiety symptoms.

Importantly, extending the extant evidence, the present study further showed that there were significant serial mediating effects of parental attachment and peer attachment in the relationships between perceived parent marital conflict and social anxiety symptoms of college students. Specifically, higher levels of perceived parental marital conflict are negatively associated with parental attachment and then peer attachment, which is related to increased social anxiety symptoms of college students. According to Davies and Cummings (1994), parental marital conflict may negatively influence attachment by increasing the negativity of parent-child relationships or by decreasing emotional availability and parental involvement, whereas secure attachment relationships might buffer the impact of parental marital conflict on adaptations of an individual. Based on EST and attachment theory, emotional distress from perceived parental marital conflict spilled over to the attachment relationships between parents and their children, which resulted in the incompetence of children in maintaining close relationships, such as peer attachment in emerging adulthood. College students who have perceived more parental marital conflict might be more vulnerable to develop low-quality attachment relationships with their parents, which in turn might be more likely to form low-quality attachment relationships with peers, thus developing social anxiety symptoms. In contrast, college students who have perceived less parental marital conflict would gain from the security of attachment with their parents, which would cultivate positive relationships in forming high-quality peer attachment, and would decrease the vulnerability in developing social anxiety symptoms. Previous studies have revealed that a higher level of perceived parental marital conflict was positively related to low-quality parental attachment relationships, which in turn might be linked to problematic peer attachment relationships of individuals (Yang et al., 2016; Tan et al., 2018), and that individuals who had formed poor attachment relationships with their parents and peers tended to report a higher level of social anxiety symptoms (Gorrese, 2016). Our study provides direct evidence showing that perceived parent marital conflict exerted its indirect effect on social anxiety symptoms through a serial multiple mediation role of parental attachment to peer attachment.

The present study has important implications. For theoretical contributions, based on the EST and attachment relationship approach, we provide important empirical evidence to explain the underlying mechanisms of how the psychosocial environment of a family (i.e., parent marital conflict) could impact social anxiety symptoms of Chinese college students, which extends the applicability of these two constructs in Chinese society. For practical implications, first, our findings indicate that parents should consciously avoid or reduce conflicts, especially in front of their children. Furthermore, they should spend more time bonding with their children, with warmth and encouragement, as high-quality attachment relationships might form a harmonious atmosphere within the family. This helps to improve marital quality and to reduce the emotional discomfort of the youths (Cox and Paley, 2003; Lindsey et al., 2009; Yang et al., 2016). Second, since peers are significant figures and contribute to multiple aspects of social adjustments of college students (Nelis and Rae, 2009; Balluerka et al., 2016; Wang et al., 2016; Yang et al., 2016), it should be recommended for families and schools to establish relevant interventions to promote and cultivate positive peer attachment relationships, which might relieve social anxiety symptoms of college students and further promote the harmonious development of communities as well as interpersonal relationships (Nelis and Rae, 2009; Balluerka et al., 2016). Third, for clinical institutions, the present findings provide guidance that healthcare professionals can be involved in mental health promotions and interventions (consider the impacts of family and peer factors in the intervention design) in college students, and brief interventions to improve self-efficacy and motivate college students to engage in healthy activities could be useful in decreasing their social anxiety symptoms (Turner and Dadds, 2001; Harold and Sellers, 2018; Sang and Tan, 2018; Yang et al., 2020). Collectively, for improving mental health level of individuals, marital quality, interpersonal relationships, clinical practice, and further promoting the harmonious development of society, our findings hold significant implications.

There are several limitations of the present study which should be considered in future studies. First, although our findings identified a positive relationship between perceived parental marital conflict and social anxiety symptoms, the crosssectional nature of our study prevented us from examining the causality of the relationship between variables. Thus, future longitudinal and experimental researches should be carried out to examine the causal direction among perceived parental marital conflict, parental, peer attachment, and social anxiety symptoms of college students. Second, data for the current study was only collected from college students. To decrease the possibility of method bias, future studies should consider multiple measures and broader samples including parents, peers, and teachers. Third, other family-related risk factors that may negatively affect social adjustment, psychological well-being, and academic achievement of adolescents should be explored in future studies. Fourth, participants were recruited *via* convenience sampling, which limits the generalizability of the study findings; future research can use additional samples to investigate the validity and repeatability of our findings.

In conclusion, the current study investigated the core family environmental factor (i.e., parental marital conflict) and attachment relationships (i.e., parental, peer attachment) in relation to social anxiety symptoms of Chinese college students. Results showed that both perceived parental marital conflict and the quality of attachment relationships were important factors associated with social anxiety symptoms of college students. Furthermore, the present study highlighted the serial multiple mediation role of parental and peer attachment in the relationship between interparental conflict and social anxiety symptoms of college students. Overall, the present findings highlighted the importance of considering attachment relationships in understanding the mechanisms linking family risk factors (i.e., parental marital conflict) to social anxiety symptoms among Chinese college students.

## Data Availability Statement

The raw data supporting the conclusions of this article will be made available by the authors, without undue reservation.

## Ethics Statement

The studies involving human participants were reviewed and approved by the Academic Ethics Committee of the School of Psychology, Shaanxi Normal University, China. Informed consent was obtained by participants prior to data collection.

## Author Contributions

ZW played the guiding role in designing research, providing suggestions for the first draft, and revision. AA and YH collected research data. AA and YZ contributed equally to this paper, for AA writing the first draft. YZ finishing the revision and improving the quality of this paper. All authors provided critical feedback and approved the final version submitted.

## Conflict of Interest

The authors declare that the research was conducted in the absence of any commercial or financial relationships that could be construed as a potential conflict of interest.

## Publisher's Note

All claims expressed in this article are solely those of the authors and do not necessarily represent those of their affiliated organizations, or those of the publisher, the editors and the reviewers. Any product that may be evaluated in this article, or claim that may be made by its manufacturer, is not guaranteed or endorsed by the publisher.
